# European food-based dietary guidelines: mapping methodological heterogeneity, sustainability integration gaps and implementation barriers

**DOI:** 10.3389/fnut.2026.1924163

**Published:** 2026-09-22

**Authors:** Vittoria Aureli, Federica Grant, Laura Rossi

**Affiliations:** 1 Council for Agricultural Research and Economics—Research Centre for Food and Nutrition (CREA—Food and Nutrition), Rome, Italy; 2 Department of Agricultural, Forestry and Food Sciences (DISAFA), University of Turin, Grugliasco, Italy; 3 Department of Agricultural, Environmental and Food Sciences (DiAAA), University of Molise, Campobasso, Italy; 4 Department of Food Safety, Nutrition, and Veterinary Public Health, National Institute of Health, Rome, Italy

**Keywords:** food groups, food-based dietary guidelines, implementation, portion sizes, sustainability

## Abstract

**Introduction:**

Food-Based Dietary Guidelines (FBDGs) are increasingly recognized as instruments to support sustainable food systems. However, substantial heterogeneity persists across Europe regarding different aspects. This paper provides a policy-oriented review of the main shortcomings affecting European FBDGs and discusses actionable recommendations to improve harmonization and effectiveness across countries.

**Methodology:**

The analysis was based on a targeted literature review and consultations with project partners and national competent authorities involved in FBDGs development. The review focused on four main areas: methodological approaches, integration of environmental sustainability, standardization issues (food groups and portion sizes), and implementation and communication gaps.

**Results:**

The findings of this study highlight marked variability across European countries in the methods used to develop FBDGs, with only a limited number of countries adopting mathematical optimization models and integrating environmental sustainability criteria (Germany, the Netherlands and Denmark). Considerable inconsistencies were also identified in food group classifications and portion size definition, limiting cross-country comparability. In addition, implementation and dissemination strategies remain fragmented, with only a few European countries reporting structured operational plans, dedicated budgets, and monitoring systems.

**Discussion:**

This work emphasizes the need for stronger European coordination to support methodological harmonization while preserving national cultural adaptation. Actionable recommendations include the development of a shared methodological framework, the integration of sustainability indicators into dietary modelling approach, the harmonization of food group and portion size terminology, and the strengthening of implementation, communication, and monitoring strategies.

## 1 Introduction

Food-based dietary guidelines (FBDGs) play a pivotal role in shaping public health policies and food environments by translating complex scientific evidence into practical recommendations for different target groups, including consumers, public institutions and food service providers (1). Traditionally, FBDGs have been developed primarily with a focus on human health, aiming to prevent nutrient deficiencies and reduce the burden of non-communicable diseases (NCDs) in the global population (2). However, the emergence of global environmental challenges such as climate change, biodiversity loss and environmental degradation and a growing recognition of the connection between health and environmental dimensions has prompted a shift towards more holistic approaches (3) in line with the One Health strategy (4) and broader sustainability and food systems perspectives.

At the global level, several international organizations have set up methodological guidance to support the development of FBDGs. Frameworks such as those proposed by European Food Safety Authority (EFSA) (5) and Food and Agriculture Organization (FAO) and World Health Organization (WHO) (2) emphasize the importance of transparent, inclusive and evidence-based processes to ensure that dietary guidelines are scientifically robust and relevant to policy. However, countries worldwide when creating national recommendations rely on heterogeneous approaches (6), which may differ considerably in terms of how scientific evidence is selected and evaluated (e.g., systematic review, expert consensus, GRADE system, etc.), and development processes (7).

In addition to these differences, further divergence lies in the extent to which environmental sustainability criteria are integrated into FBDGs. One of the most recent reviews on the topic, carried out by James-Martin et al. (8) found that only 37 dietary guidelines worldwide out of 83 studied include environmental sustainability considerations, which were often framed as general advice with limited actionable or quantifiable recommendations. Similarly, Martini et al. (6) emphasized that, while current FBDGs address health-related aspects, they require revisions and improvements to fill gaps concerning guidance on the environmental impacts of diets. These discrepancies are largely associated with the lack of a clear methodology for integrating environmental criteria into dietary guidelines (3).

Moreover, the lack of alignment in food group standardization and portion size quantification represents an additional source of heterogeneity among dietary guidelines at the global level (9–11). Although food groups are generally intended to reflect shared nutritional characteristics and public health relevance, their classification varies substantially both at the global level and across countries within the same continent, driven by differences in nutritional priorities, cultural practices, and methodological approaches (9, 12). Similar challenges are observed due to the absence of a harmonized quantification for portion sizes and a common terminology across the guidelines (9). Taken together, all these differences substantially limit the comparability of dietary guidelines at the global level and within regions.

In addition to this methodological heterogeneity, the effectiveness of FBDGs is also influenced by challenges in their implementation and communication, which may limit their capacity to influence consumers’ dietary behaviors. At European level, guidance documents such as those developed by EFSA emphasize that a clear implementation plan is a prerequisite for the long-term effectiveness of dietary guidelines (5). However, the study of Wijesinha-Bettoni et al. (13), found that 24 countries out of 27 surveyed worldwide have a body responsible for implementation plans, only 11 have a dedicated budget, and 7 have established monitoring systems. Additionally, in terms of the capacity to reach consumers as the final end-users, several challenges persist in effective dissemination and communication of FBDGs. As stated by Amorim et al. (14), to avoid confusing the target audience, FBDGs should be presented as clearly, accurately, and practically as possible. However, translating complex scientific evidence into clear and practical messages for non-expert audiences remain difficult, particularly when recommendations and the overall complexity of guideline materials may further hinder their understanding (15, 16).

Building on previous work (17) conducted within the framework of the EU Horizon project PLAN’EAT,^1^ which compared the FBDGs of eleven European countries (Belgium, France, Germany, Greece, Hungary, Ireland, Italy, Poland, the Netherlands, Spain and Sweden), this study extends the analysis to European Countries for which relevant secondary data and official documentation were available. By covering evidence and secondary data available across European countries, this study provides a broad assessment of selected characteristics of European FBDGs, identifying common patterns, methodological differences, and opportunities for greater harmonisation while preserving national adaptations. Hence, the first objective is to explore methodological characteristics, including development processes, the incorporation of environmental sustainability criteria, and issues related to standardization of food groups and portion size definition and quantification. The second objective is to examine implementation, dissemination, and communication practices, highlighting structural gaps, inconsistencies, and areas requiring better coordination.

Existing reviews have examined several important dimensions of FBDGs, including the integration of sustainability (6, 8), methodological approaches to guideline development (18, 19), food group classification and portion size definitions (9–12), and implementation or dissemination aspects (13, 20, 21). While these studies have significantly advanced the field, they generally address individual components of FBDGs in isolation. In contrast, this study adopts an integrated European perspective that combines methodological approach, sustainability, standardization, and implementation considerations within a common framework. By examining these shortcomings in terms of interconnected dimensions, this study seeks to provide an evidence-informed overview of current divergences and identify practical directions to enhance the coherence, effectiveness, and policy relevance of FBDGs across Europe.

## 2 Methodological framework

This work began with a preliminary consultation involving 10 partners of the PLAN’EAT project representing different disciplinary backgrounds, including nutrition, economics, sustainability, and public health, aimed at refining the scope of the analysis and identifying the main shortcomings of the FBDGs in European countries for which secondary data were available. The experts were based in Italy, Ireland, Germany, France, and Denmark and represented a range of organisations, including universities, public research institutions, and private organisations, ensuring a multidisciplinary and cross-sectoral perspective throughout the consultation process. Discussions were conducted through dedicated project meetings and structured consultations.

Based on these exchanges, four main key areas for analysing FBDGs were identified: (1) which methodological approaches and evidence-based processes are adopted for FBDGs development? (2) how health and environmental sustainability criteria are incorporated in FBDGs?; (3) to what extent do key components of FBDGs, particularly food group classifications and portion size definitions, vary across European countries? (4) what strategies are in place for the implementation, communication and dissemination of FBDGs? These themes constituted the analytical framework used for the subsequent review and assessment of European FBDGs. The identified areas were further explored through a targeted literature review, combined with consultations involving European experts outside the PLAN’EAT consortium.

The analysis of these thematic areas highlighted four major areas of divergences across existing FBDGs: (I) methodological approaches to guidelines development, (II) integration of environmental sustainability criteria, (III) food group classifications and portion size definitions and (IV) implementation, communication and dissemination strategies. A policy document on which this work is based reports the outcomes of this analysis (22). While the policy document reports the main outcomes of this process and provides recommendations specifically for policymarkers, the present work extends beyond its policy-oriented scope. It provides a scientific synthesis of the available evidence by integrating findings from the literature review, expert consultations and policy perspective. In this way, this study offers a broad overview of existing limitations while highlighting opportunities for future harmonization across countries. Figure 1 summarizes the methodological framework adopted for the study and the different steps.

**Figure 1:**
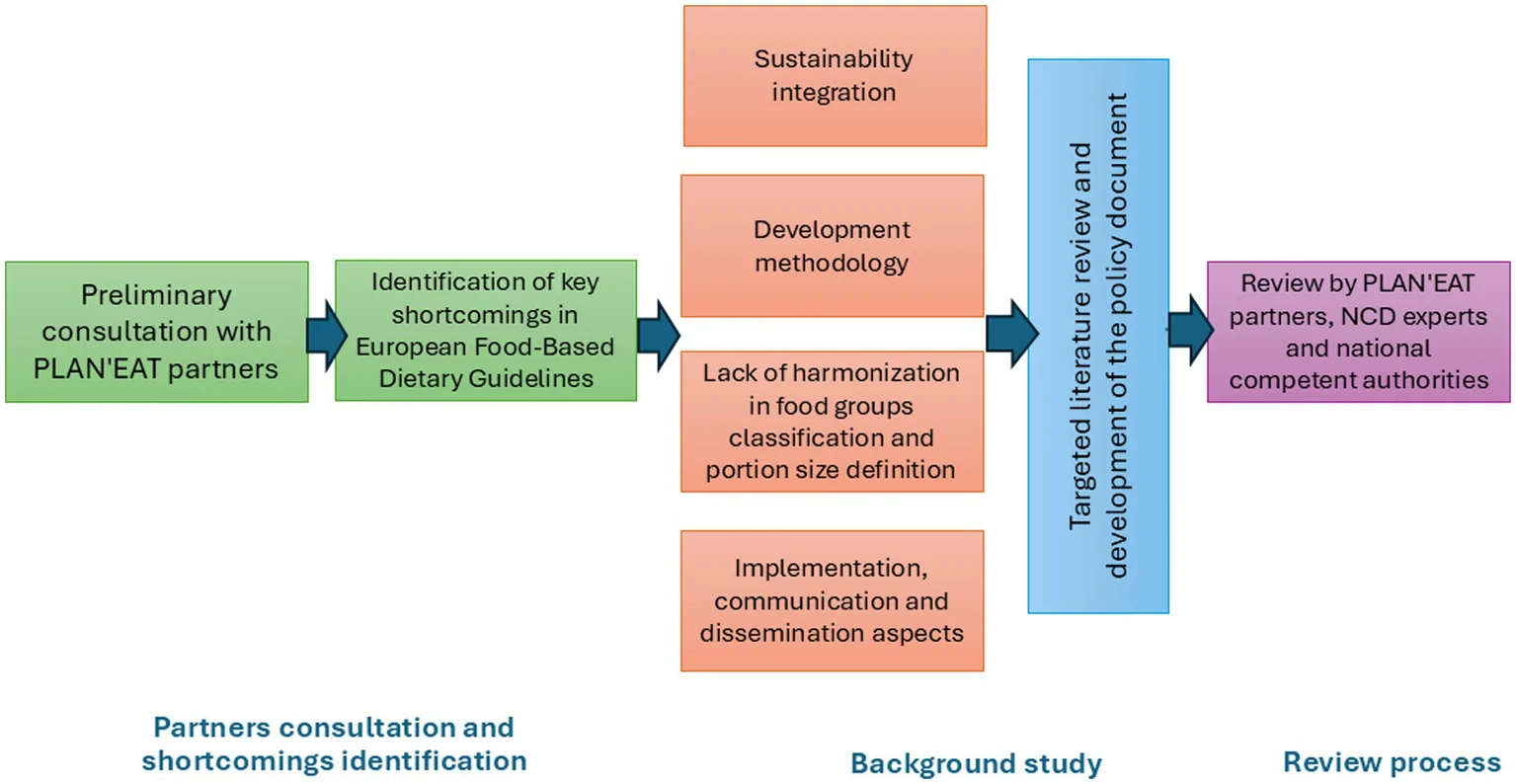
Development process of the methodological framework, including partner consultation, identification of shortcomings in existing European FBDGs, targeted literature review, document development, and external review and validation.

### 2.1 Literature research

The targeted literature search was conducted between October and November 2024 using scientific databases PubMed, Scopus and Google Scholar to retrieve publications and international reports related to the analysis of the different shortcomings in FBDGs, identified by the experts. The core terms “FBDGs”, “food-based dietary guidelines”, “dietary guidelines” were combined using Boolean operators (AND/OR) with terms corresponding to each anlytical domain, including “sustainable diets”, “environmental sustainability”, “food groups”, “portion sizes”, “implementation”, “communication”, “dissemination”, and “Europe” or “European.” For example, search combinations included “food-based dietary guidelines” AND sustainability and “dietary guidelines” AND implementation AND Europe. Peer-reviewed articles, reports from international organisations, and policy documents published in English were eligible when they provided methodological, descriptive, or comparative information on European national FBDGs relevant to at least one of the four analytical domains: guideline development, sustainability integration, food group/portion size standardization, or implementation and communication. Documents that did not address national FBDGs or did not provide information relevant to any of these domains were excluded. The eligible collected documents were screened independently by two authors (VA and FG). Any uncertainty regarding eligibility or disagreements between reviewers were discussed and resolved through consensus. When necessary, a third author (LR) was consulted to arbitrate and reach a final consensus. Additional searches were performed for each of the four shortcomings identified during the preliminary consultation. The final literature review was carried out between February and June 2026, to reinforce the policy document with the most recent scientific evidence. Peer-reviewed articles, reports from international organizations, and policy documents published in English were considered eligible for inclusion if addressing the development, implementation, sustainability integration, or evaluation of FBDGs. Documents not addressing these topics were excluded. Information extraction was conducted by the same two authors (VA and FG) using the four analytical domains identified during the expert consultation. When multiple sources reported different or incomplete information, discrepancies were resolved through comparison across sources and discussion among the authors. Additionally, information on national FBDGs was integrated, when available, consulting the European Commission’s Knowledge4Policy platform (23) enabling coverage across European countries. Information retrieved from this platform was used to complement and verify evidence identified through the literature review where relevant. Similarities, differences, and methodological challenges across European FBDGs were subsequently identified and synthesized according to the four domains defined through the preliminary expert consultation. As the present analysis relied on secondary evidence rather than on a direct country-by-country assessment of all national FBDGs, geographical coverage varied across analytical domains according to the scope of individual sources and the availability of country-specific information. Therefore, the countries represented in the evidence base differed across the methodological, sustainability, food-group classification, portion-size, and implementation/communication domains. Country-level statements reported in the Results should consequently be interpreted as referring to the countries included in the respective cited sources, rather than to the full set of European countries. Additionally, given the heterogeneous nature of the evidence base and the policy-oriented scope of the study, no formal assessment of study quality was conducted. Findings reported throughout the manuscript derive either from previously published evidence or from the authors’ synthesis of information collected across multiple sources.

### 2.2 Expert consultation and policy document development

The drafting of the policy document followed a multi-step approach. Initially, evidence from the literature searches and prior PLAN’EAT project outputs was synthesized into a preliminary draft to systematize the information and identify similarities and inconsistencies across FBDG components. The draft was then reviewed internally by project partners to ensure scientific consistency. Subsequently, to strengthen institutional robustness, the policy document was reviewed by NCD experts, including two clinicians involved in the PLAN’EAT project and four registered dietitians external to the project, together with national competent authorities responsible for national FBDGs in nine of the eleven PLAN’EAT participating countries (Belgium, France, Germany, Hungary, Ireland, Italy, Poland, the Netherlands, and Sweden). Based on comments emerging from the consultations, improvements and further refinements have been incorporated into the policy document.

## 3 Assessment of guidelines options and implications

The development of European FBDGs is characterized by a substantial heterogeneity in methodological approaches, and such variability may limit comparability across countries. The following subsections examine methodological shortcomings, the integration of environmental sustainability dimensions, and the issues related to the standardization of food groups and portion sizes. Because the evidence base differed across analytical domains, the countries represented in the following subsections are not identical. Country-specific findings are therefore reported in relation to the scope of the respective sources cited.

### 3.1 Methodological shortcomings in the development of FBDGs

The analysis conducted by Salesse et al. (18) pointed out that dietary recommendations in Europe are generally developed through literature reviews and expert consensus. In some cases, these approaches are complemented by simplified quantitative calculations considering different energy requirements and selected demographic variables, such as gender (18).

Based on the available secondary evidence, mathematical optimization approaches for the development of FBDGs have been documented in five European countries: Denmark, France, Germany, the Netherlands, and the United Kingdom (24–27). Such models minimize or maximize predefined objective functions, such as macronutrient and/or micronutrient intake, total energy intake, or Green House Gas (GHG) emissions, while adhering to established constraints, including dietary reference values (DVRs), or environmental sustainability thresholds (28).

Among countries employing diet-modelling systems, both commonalities and substantial divergences are observed. All five countries rely on quantitative optimization approaches to identify dietary patterns that align with national nutritional requirements while minimizing deviations from actual populations’ food consumption (29). However, the extent to which sustainability criteria are incorporated into these modelling approaches varies significantly. In particular, France and the UK apply advanced modelling techniques based on linear programming primarily focused on nutritional and health optimization without including environmental sustainability constraints. In France, the Agence nationale de sécurité sanitaire (ANSES) developed Healthy Eating Patterns (HEPs) using a linear programming framework that simultaneously integrated multiple dimensions of diet quality, including nutrient adequacy, chronic disease prevention, dietary acceptability, and food safety through contaminant exposure constraints (30). Similarly, the UK Eatwell Guide was developed using a linear programming approach that optimised food group proportions to meet dietary reference values and food-based recommendations while maintaining diets as close as possible to current consumption patterns (31). By contrast, countries such as the Netherlands, Germany and Denmark also integrate sustainability aspects into their modelling approach, as discussed in the following section.

### 3.2 Integration of environmental sustainability in FBDGs

Different European countries include recommendations related to some aspects of environmental sustainability in their dietary guidelines with varying grades of specificity. According to the European Commission Knowledge4Policy repository (32), sustainability-related dietary guidance was identified for 23 European countries included in the platform. According to the latest revision of the Nordic Nutrition Recommendations (33), Denmark, Finland, Iceland, Norway, and Sweden share a strong sustainability-based strategy, which emphasizes reduced consumption of meat and dairy products, increased intake of plant-based foods, and alignment of food production with local capacities and emerging food technologies. Beyond these qualitative sustainability-oriented recommendations, some European countries have applied modelling approaches to integrate sustainability criteria into FBDGs. Germany, the Netherlands, and Denmark represent notable examples, although the approaches adopted differ substantially. Germany includes directly GHGEs and land use as environmental indicators (27), whereas the Netherlands incorporates environmental considerations indirectly by imposing maximum limits on the consumption of animal-based foods associated with higher environmental impacts (e.g., red meat and total meat) (25). Denmark adopts a different approach, where environmental sustainability is incorporated including the EAT-Lancet reference diet (34) into the optimization model (26). In addition, Italy (35) and Switzerland (36) include qualitative sustainability guidance, encouraging plant-based foods consumption, seasonal choices, and reduction of red and processed meat and food waste. Other countries such as Belgium, Spain, France, Estonia, Lithuania, Luxembourg, Poland, and Slovenia provide qualitative rather than quantitative sustainability-oriented recommendations (32).

Overall, the results of this analysis presented in Figure 2 indicate a spectrum of sustainability integration across Europe, ranging from advanced modelling approaches to broader general recommendations, reflecting heterogeneity in national methods.

**Figure 2:**
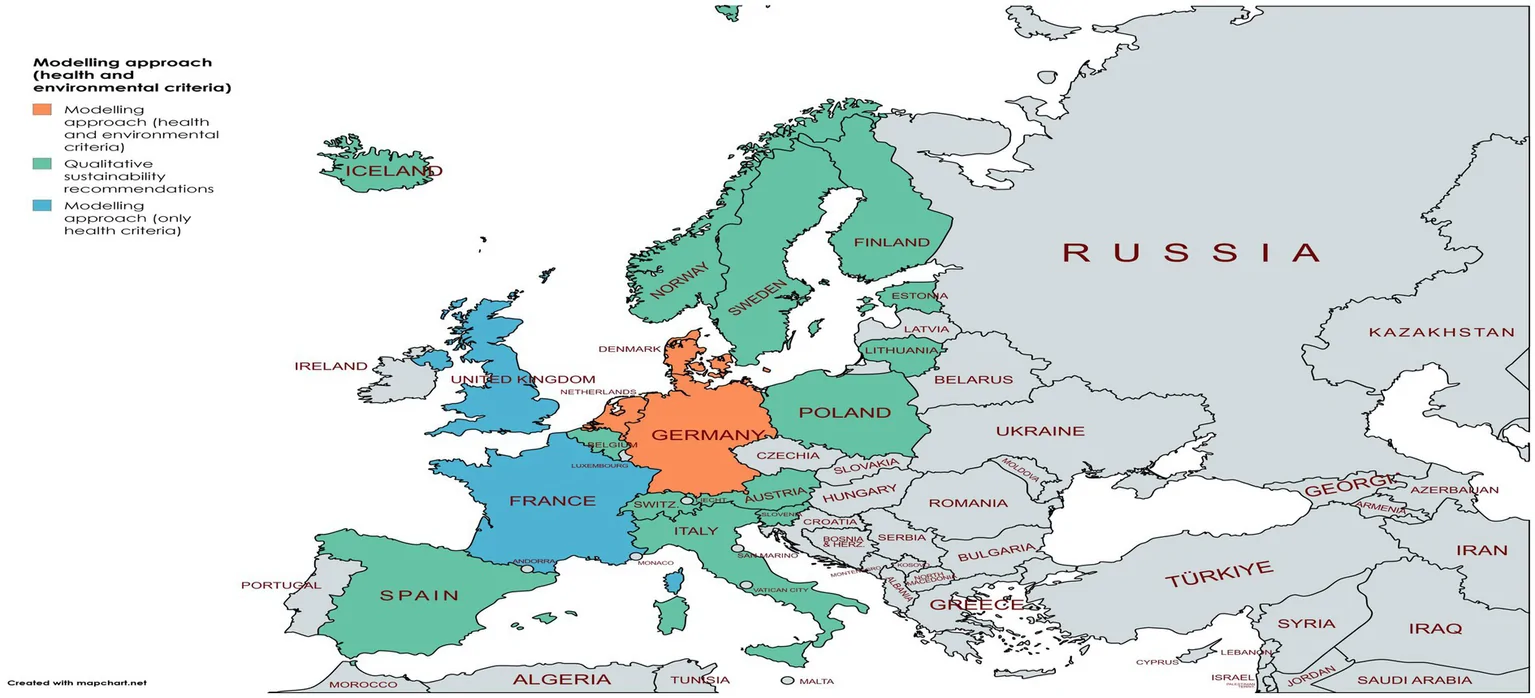
The figure highlights countries adopting modelling approaches for the development of FBDGs, distinguishing between those that integrate quantitative health and environmental sustainability criteria (orange), those that include only quantitative health criteria and no sustainability information (blue), and those that include a primary focus on health aspects and only general qualitative recommendations on environmental sustainability (green). Countries highlighted in yellow indicate the absence of sustainability aspects in their FBDGs or the unavailability of relevant data. Created with mapchart.net, licensed under CC BY-SA 4.0.

### 3.3 Standardization issue: food groups and portion sizes

#### 3.3.1 Food groups

A food group is defined as a set of foods sharing a common origin in primary production and/or similar nutrient composition (37). According to Herforth et al. (12), fruits and vegetables were grouped consistently in approximately 85% of the European FBDGs included in their analysis, whereas greater variability was observed for protein-rich and animal-source foods The study by Hughes et al. (38) analyzed the inclusion of legumes within different food groups in FBDGs worldwide. Considering only the European food groups classification, legumes are mostly included in the fruit and vegetables group in Austria, Finland, and Hungary; in the vegetables group in Estonia and Germany; and in the protein-rich foods group in Albania, Belgium, Bulgaria, Iceland, Ireland, Latvia, Malta, the Netherlands, Slovenia, Spain, Turkey, and the UK.

Similarly, nuts are inconsistently categorized across FBDGs. Some countries classify nuts within the protein-rich food group (e.g., Latvia, Ireland, Bulgaria, Spain, and Malta), while others place them in the fats and oils group due to their high fat content (e.g., Switzerland and Estonia) (21), reflecting variability in how their nutritional role is conceptualized within national dietary frameworks.

Another issue identified relates to the grouping of foods with heterogeneous nutritional profiles into a single food category, alongside undifferentiated general recommendation. Herforth et al. (12) reported that dairy products were presented as a separate food group in approximately 85% of the European FBDGs included in their analysis. Dairy products resulted grouped with animal proteins only in Estonia, Latvia and Switzerland as reported by Montagnese et al. (21). The dairy group generally includes foods such as milk, yogurt, and other fermented dairy products recommended for daily consumption to ensure adequate calcium, protein, and vitamin intake, and cheese with a different dairy-fat matrix recommended at lower consumption (39). More than half of European FBDGs recommend 2–4 servings of dairy per day, and over 80% include specific messaging on cheese, generally emphasizing low-fat or fat-free options, fermented products, and a variety of fresh cheeses (40). However, only few European countries specify a daily recommendation for milk or yoghurt (e.g., Cyprus, Hungary, Italy, Poland) (18).

#### 3.3.2 Portion sizes

Connected with the issue of the food groups is the aspect of portion sizes’ definitions showing large variability and elements of divergencies. While the term “portion” generally refers to the amount of food consumed on a single eating occasion, its interpretation varies widely across countries (41). In food labelling, this term denotes the amount of food reasonably expected to be consumed in one occasion and is usually presented as a “serving” on nutrition labels (42). These heterogenous definitions can lead consumers to misinterpret the labeled serving as a recommended portion rather than an average consumption unit (43). In addition to the absence of a shared terminology, a substantial degree of variability was observed across FBDGs in terms of size as stated in the different studies. Salesse et al. (18) reported that portion sizes for comparable foods vary widely across and within world regions, with European guidelines offering some of the highest median portion sizes for meat, fish, and legumes. Similarly, Carruba et al. (10), in their comparative analysis of 34 European national FBDGs, reported considerable variability in standard portion sizes across the countries included in their study, particularly for staple foods such as bread, pasta, milk, and vegetables indicating heterogeneous national interpretations of the same food categories. Moreover, the study showed that European FBDGs differ in the recommended number of daily portions, the gram weights used to define a portion, and the level of detail provided to consumers (10). In line with these findings, recent evidence further highlights marked heterogeneity in the quantification of serving sizes in worldwide FBDGs, including differences in units of expression (e.g., grams versus household measures), levels of numerical precision, and overall standardization across national guidelines (11).

### 3.4 Implementation, communication and dissemination aspects

Implementation refers to the comprehensive process of integrating FBDGs into national policies, programs, and at multi-sectoral levels to influence diets and the food system (44). Nevertheless, numerous barriers limit the effective implementation of European FBDGs. The study of Wijesinha-Bettoni et al. (13), reported the results of a key informant survey carried out in 27 countries worldwide highlighting that most countries have an official body responsible for dietary guidelines’ implementation. Within the European countries included in the mentioned study (13), only Denmark, Finland, Germany, Malta and Norway reported a defined strategy or an operational plan. Furthermore, only Denmark, Finland, Malta and Norway indicated the presence of a dedicated budget covering both dissemination and implementation activities, whereas Germany, Iceland and Sweden reported funding allocated solely to dissemination strategies. Systematic monitoring and evaluation (M&E) mechanisms were reported only in Denmark, Finland, Germany and Norway (13). Beyond this, several actions have been proposed to support the implementation of FBDGs, including the integration of dietary guidelines into school meal standards, public food procurement policies, workplace catering services, nutrition education programs, and healthcare setting and other relevant food environments (13, 44–46). In addition, interventions targeting food environments, such as increasing the availability and accessibility of healthier foods, price incentives and food promotion strategies have shown potential to support healthier and more sustainable food choices and improve adherence to dietary recommendations (47–49). These results align with Keller and Lang (45), highlighting that insufficient financial resources and challenges in reaching vulnerable populations (e.g., low-income people) are key barriers to the effective implementation of FBDGs.

Beyond implementation barriers, effective communication and dissemination activities remain a major challenge. According to WHO’s strategic communications framework (50), public health messages should be accessible, actionable, credible, trustworthy, relevant, timely, and easily understandable. However, a report from the Federation of European Nutrition Societies (51) has identified several limitations related to the communication of European FBDGs including their frequent lack of translation into clear and actionable messages for consumers, insufficient dissemination strategies, limited tailoring of communication to different target groups, and the overall complexity of the information provided.

To facilitate the dissemination of FBDGs, most European countries include a specific graphical representation to summarize and explain the recommendations. Nevertheless, heterogeneity can be highlighted again. Food pyramids represent the most frequently used format (e.g., Austria, Belgium, Bulgaria, Croatia, Cyprus, Czech Republic, Finland, Greece, Latvia, Lithuania, Luxembourg, Poland, Romania, Slovenia and Spain), although some countries adopt alternative representations such as wheel-shaped formats (Germany and Sweden), a plate (Finland) or a house (Hungary) (52). In addition, European graphical representations differ not only in format but also in structure and level of detail as stated in Montagnese et al. (21). The number of food groups and hierarchical levels included varies considerably across countries, reflecting differences in how dietary messages are prioritized and communicated (21). This variability may limit the comparability of communication approaches across countries and reflects differing national strategies for translating dietary recommendations into consumer tailored messages.

## 4 Actionable recommendations

The recommendations presented below reflect a combination of evidence identified in the targeted reviewed literature, expert consultations, and the authors’ interpretation of the findings. They should therefore be considered as policy-oriented options for discussion rather than prescriptive recommendations.:

- Establish a European common methodological framework for FBDGs development, based on mathematical modelling approaches with shared core data inputs (e.g., harmonized food consumption data, nutrient reference values, and environmental indicators) while allowing national contextual adaptation. Compared to the EFSA approach, this framework would place greater emphasis on modelling and the integration of sustainability criteria alongside nutritional adequacy. Such framework would address key methodological limitations of current approaches, including heterogeneous development processes, inconsistent food group classifications, and varying portion size definitions, fostering a more coherent, interoperable, and robust harmonization across European countries.
- Promote the progressive integration of measurable sustainability criteria into dietary modelling approaches moving from qualitative sustainability advice to quantified and operational targets. Potential approaches could include Life Cycle Assessment or emerging methodology such as True Cost Accounting.
- Develop a European reference taxonomy for food group classification to enhance cross-country comparability while preserving cultural specificity.
- Harmonize the technical distinction of “portion” (recommended intake), differentiating it from the term “serving” (labelling unit) and introduce standardized gram-based reference portions within general food groups where applicable, to improve surveillance and research alignment.
- Consider the potential establishment of a European coordination body for FBDGs, similar to the role played by EFSA in harmonizing food consumption data through EU Menu- Such mechanism could support member states in designing and developing implementation strategy planning, collecting best practices that can be replicated in other countries and providing experts to address national capacity gaps.
- Promote the development and validation of European dietary adherence indices adapted for national FBDGs, allowing systematic assessment of populations compliance with the dietary recommendations, which can inform the efficacy of the implementation strategies.
- Strengthen communication strategies through messaging that clearly explains the rationale behind dietary recommendations, the recommended actions, and how to apply these recommendations in everyday life, culturally adapted, and audience-specific messaging, with particular attention to vulnerable and low socio-economic population groups.
- Strengthen the integration of FBDGs into national policies and implementation programmes across sectors, promoting their adoption as reference standards in public food environments and institutional settings, such as school canteens, educational programmes, and workplace catering services.

To facilitate the interpretation of the findings and the corresponding recommendations, Table 1 provides a summary of the main shortcomings identified across European FBDGs and the proposed actions to address them.

**Table 1:** Summary of key shortcomings identified in European FBDGs and corresponding actionable recommendations.

Domain	Main shortcomings identified	Proposed actions
Development methodology	Heterogeneous approaches to evidence assessment and guideline development; limited use of optimization models	Develop a common European methodological framework based on shared data inputs and modelling principles
Sustainability integration	Sustainability often addressed qualitatively; limited incorporation of environmental indicators	Progressively integrate measurable sustainability criteria using standardized assessment approaches
Food group classification	Inconsistent categorization of foods across countries	Develop a European reference taxonomy while preserving national specificities
Portion size definition	Differences in terminology, recommended servings (frequencies), and quantities	Harmonize terminology and establish standardized reference portions
Implementation and dissemination	Limited operational plans, dedicated budgets, monitoring systems, and dissemination strategies	Strengthen implementation plans, promote knowledge exchange, develop impact assessment indicators, and support capacity building
Communication	Complex messages and insufficient tailoring to target groups (e.g., sex-differentiated recommendations, vulnerable individuals, etc.)	Develop audience-specific communication strategies and improve the usability of recommendations for vulnerable groups

## 5 Discussion

While FBDGs have been framed as tools to promote public health and reduce the impact of NCDs for a long time, their role is progressively expanding towards a more holistic perspective that integrates health and sustainability dimensions, and food system considerations (7). This evolution has also raised the question of whether traditional FBDGs are still sufficient, or whether countries should adopt food systems–based dietary guidelines that integrate public health goals with environmental, economic and socio-cultural dimensions of sustainability (1).

However, as already reported in the study of Aureli et al. (17), despite the growing emphasis on integrating sustainability aspects beyond a purely health-focused perspective, substantial heterogeneity across European FBDGs can be highlighted. This divergence is specifically related to technical development processes, sustainability criteria integration, food group classification, portion size definition, and implementation and communication strategies. Although these diversities may reflect legitimate differences in national cultural contexts and public health priorities, the degree of fragmentation observed in this study should be overcome in order to facilitate comparability across countries and the effectiveness of FBDGs as public health and sustainability tools at European level. Common food and nutrition policies, translated into FBDGs harmonized messages, could counteract the actual low adherence to recommendations (53), particularly among young people and specific demographic groups (for example men tend to have lower adherence than women) (17, 54, 55).

The present study highlighted that a first major area of divergence concerns the methodological approaches underlining FBDGs development (17, 19, 21). In Europe, only 5 countries (Denmark, France, Germany, The Netherlands and UK) rely on advanced modelling approaches with the aim to allow an explicitly balance between the nutritional adequacy of the diet and the general population’s current food consumption. In some cases (Denmark, Germany and the Netherlands), environmental constraints are integrated within the optimization models, increasingly aligning nutritional recommendations with environmental sustainability dimension. However, even among countries adopting such models, differences persist in the choice of health and/or environmental constraints, the modality in which these constraints are integrated in the model, and in the population food consumption dataset used. This heterogeneity and the absence of a shared methodological framework at European level limits the comparability of recommendations and complicates joint monitoring efforts. One possible strategy to overcome this problem could be to develop a European methodological common standard framework with a clear definition of health and environmental input data (e.g., EFSA food consumption data, nutrient reference value, environmental indicators, etc.), adaptable at the national level. This methodological framework could be based on modelling approaches already implemented in Germany (27), the Netherlands (25) and Denmark (26), where health and environmental constraints are clearly incorporated. The inclusion in the modelling approach of common quantitative criteria for the environmental dimension would help to address the heterogeneity currently observed across European FBDGs, in which environmental aspects are predominantly addressed through general qualitative recommendation. However, the selection of environmental sustainability indicators remains a major methodological challenge. While GHG emissions are the most frequently used metric in modelling studies, other indicators (such as land use, water use, eutrophication potential, biodiversity impacts, etc.) are also relevant for evaluating the sustainability of populations dietary patterns (3, 49). Another important aspect is related to the fact that improvements in one environmental indicator do not necessarily translate into improvements in others, and trade-offs may occur between environmental outcomes depending on the dietary scenarios considered as highlighted in Baishiri et al. (56). The relative importance of these indicators may also vary according to regional environmental priorities and resource constraints (56), as countries may be affected by different environmental challenges. Therefore, future methodological frameworks for FBDGs should promote a multidimensional assessment of environmental sustainability relying on a specific indicator adapted at regional level (17).

Variability in food group classification and portion size definitions may also contribute to consumers’ misunderstanding and may hinder cross-national comparisons in dietary surveillance, practical food and nutrition polices (e.g., food labeling), and research (10, 11, 18). While some variability is expected due to cultural and traditional dietary diversity, the absence of a shared classification undermines the comparability of FBDGs at European level and limits a pan-European food and nutrition system approach. A harmonization of food groups’ classification could facilitate cross-country analyses, allowing them to reflect specific cultural contexts. Similarly, aligning portion sizes definitions could improve consistency in dietary assessment, surveillance, and research comparability, ensuring that FBDGs provide clear guidance to diverse populations.

Furthermore, the results presented in this study underscore persistent challenges beyond technical development problems in implementation, communication, and dissemination of European FBDGs. Only a few European countries have defined strategy and/or operational plans, and fewer allocate dedicated funding for both implementation and dissemination plans (13). At the same time, systematic monitoring and evaluation mechanisms are limited, suggesting that the potential impact of FBDGs may not be fully realized. Evidence suggests that effective implementation requires more than dissemination activities alone and should be supported by a combination of policy-based measures (13). Examples include the integration of dietary guidelines into school meal programmes, public procurement policies, nutrition education initiatives, and interventions aimed at improving the availability and accessibility of healthier food options. Recent evidence also indicates that multi-level approaches targeting both individual behaviors and food environments are more likely to promote sustained dietary change than information-based interventions alone (48, 49). At the same time, several barriers hindering effective implementation were identified. These include insufficient fundings, conflicts of interest, structural and socio-economic factors, availability, affordability, and accessibility of healthy foods together with traditional and sociocultural influences (42, 43, 45). These factors may further complicate and decrease the possibility of integrating FBDGs into successful national programmes aimed at changing population dietary behaviors.

The partial or complete absence of structured implementation and dissemination plans in European countries highlights the potential value of a European coordination body for FBDGs. Such a coordination body could provide a platform for sharing best practices, assisting countries in designing and operationalizing implementation strategies, and supporting them with necessary additional experts. This kind of organization could also promote knowledge exchange and greater methodological consistency across countries. Facilitating the communication and dissemination of FBDGs could represent an additional responsibility of the coordinating body, particularly given the current challenges in effectively reaching the public and the need to strengthen and tailor nutrition messages to different audiences. Different studies indicate that many national initiatives lack formal communication plans and provide limited guidance on how to effectively convey messages to the general population as the end user, including how to tailor recommendations for different vulnerable groups (e.g., low-income group) (20, 57). However, the effective implementation of FBDGs also depends on their translation into food environments, which requires the engagement of key intermediary stakeholders (e.g., school and workplace canteens, retailers, and food service providers), who directly influence the accessibility and availability of food. The limited involvement of sectors beyond nutrition and healthcare, particularly agriculture and food production, further constrains the potential impact of FBDGs. This highlights the need for coordinated, multisectoral approaches operating across multiple policy levels to enhance their implementation and effectiveness (13, 45, 46).

Finally, the lack of validated dietary indices aligned with national guidelines limit the ability to assess population adherence to FBDGs within and across countries and to evaluate the effectiveness of implementation and of the dissemination and communication strategies. This gap could be filled both building on existing dietary adherence indices across Europe [e.g., Norwegian Dietary Guideline Index (NDGI) (58), Adherence to Italian Dietary Guidelines Index (AIDGI) (59), Programme National Nutrition Santé Guidelines Score (PNNS-GS) (60)] and validating a European dietary adherence index, that can be adapted to national FBDGs. This action may provide relevant and comparable monitoring data for policymakers and public health authorities to refine strategies and monitor progress over time.

The aim of this study was to map existing heterogeneity of dietary guidelines in European countries as highlighted in other studies (6, 8, 10–12, 18, 21) and identify practical proposals where structured coordination could improve comparability, effectiveness, and policy impact rather than prescribing a single model to be applied in all European countries. Dietary guidelines should maintain their country specificity to account for cultural, dietary, and epidemiological differences across populations, which may not be adequately captured by a fully standardized wide-regional European dietary guideline. Differences in food traditions, food availability, culinary practices, environmental conditions, and public health priorities are key elements of national recommendations’ development. The objective of harmonisation should not be to eliminate country-specific adaptations, but rather to promote greater methodological consistency, transparency, and comparability across Europe. Given that European FBDGs share more common features than differences (21), greater alignment could be achieved while preserving the flexibility required to accommodate national contexts, cultural traditions, and population needs. Based on the interconnection of methodological, implementation, and communication gaps, a centralized European coordination mechanism would represent a structural enabler of harmonization and not merely an administrative solution. However, its governance structure, resource requirements, institutional responsibilities, and potential limitations would require further evaluation before such an approach could be considered at the European level. The ambition of future research on the topic should focus on developing shared methodological approach and evaluating their feasibility and acceptability within different national contexts, ensuring that harmonization strengthens, rather than overriding, cultural and dietary diversity across Europe. Beyond the challenges identified through the analysis, an additional emerging issue that could be considered in future FBDG revisions concerns the role of novel food sources. Although this topic was beyond the scope of the present review, it may warrant further investigation given its potential implications for the sustainability of future food systems (61, 62).

## 6 Limitations

This study has some limitations that should be acknowledged. First, the analysis was based on a targeted rather than a systematic literature review and therefore may not have captured all relevant publications on European FBDGs. In addition, the availability, methodological rigor, and completeness of information varied across countries and sources, which may have influenced the level of detail available for some topics and geographical areas. Second, although information from the European Commission’s Knowledge4Policy platform was used to complement the analysis, country coverage depended on the availability of published evidence and official documentation at the time of data collection. Third, the study relied primarily on secondary sources, including scientific publications, reviews, reports, and policy documents, rather than on a direct assessment of individual national FBDGs. The heterogeneous geographical scope of the underlying sources also limits direct cross-country comparisons. Absence of information for a given country in a specific analytical domain should not be interpreted as evidence that the corresponding feature is absent from its national FBDG, but rather as reflecting the scope and availability of the secondary evidence included in this review. Additionally, the analysis did not evaluate the extent to which existing FBDGs provide population-specific recommendations tailored to factors such as age, sex, physiological status, lifestyle, or physical activity level. Finally, the identification and classification of shortcomings involved expert judgement and evidence synthesis, which may have introduced a degree of subjectivity despite the iterative consultation process involving both PLAN’EAT partners and external experts. While this approach helped focus the analysis, it may have influenced the identification, selection and interpretation of evidence by prioritizing some aspects over potentially relevant others. These limitations should be considered when interpreting the findings and recommendations presented in this work.

## Footnotes

1
https://planeat-project.eu/
